# Beyond HbA_1c_: Environmental Risk Factors for Diabetic Retinopathy

**DOI:** 10.4172/2155-9570.1000405

**Published:** 2015-03-13

**Authors:** Kristen Harris Nwanyanwu, Paula-Anne Newman-Casey, Thomas W Gardner, Jennifer I Lim

**Affiliations:** 1 University of Illinois at Chicago, Chicago, Illinois, USA; 2 University of Michigan, Ann Arbor, Michigan, USA

**Keywords:** Diabetes, Diabetic retinopathy, Retina

## Abstract

Diabetic retinopathy affects 4.2 million people in the United States and is the leading cause of blindness in working-aged people. As the prevalence of diabetes continues to rise, cost-effective interventions to decrease blindness from diabetic retinopathy will be paramount. While HbA_1c_ and duration of disease are known risk factors, they account for only 11% of the risk of developing microvascular complications from the disease. The assessment of environmental risk factors for diabetic eye disease allows for the determination of modifiable population-level challenges that may be addressed to facilitate the end of blindness from diabetes.

## Introduction

Diabetic retinopathy is a multi-factorial condition for which the pathophysiology is incompletely understood. Eleven percent of the risk of developing microvascular complications related to diabetes may be attributed to HbA_1c_ and duration of disease, which leaves 89% to be determined [1]. Approximately 4.2 million people in the United States of America (US) have diabetic retinopathy (DR), of which 655,000 have vision-threatening proliferative diabetic retinopathy or macular edema [2]. According to the Centers for Disease Control and Prevention, the US spends $500 million annually on diabetes-related blindness [3]. Despite known, effective treatment for DR, it remains the leading cause of blindness in working-aged people in the US. Diabetic eye disease is more likely to develop in the Non-Hispanic Black population, in Latinos, in Native Americans, and in patients with poor socioeconomic status [4–8]. While individual risk factors have been determined, characteristics of the communities in which patients live have also been found to influence outcomes in overall health [9] and in diabetes in particular [10].

**Figure 1:**
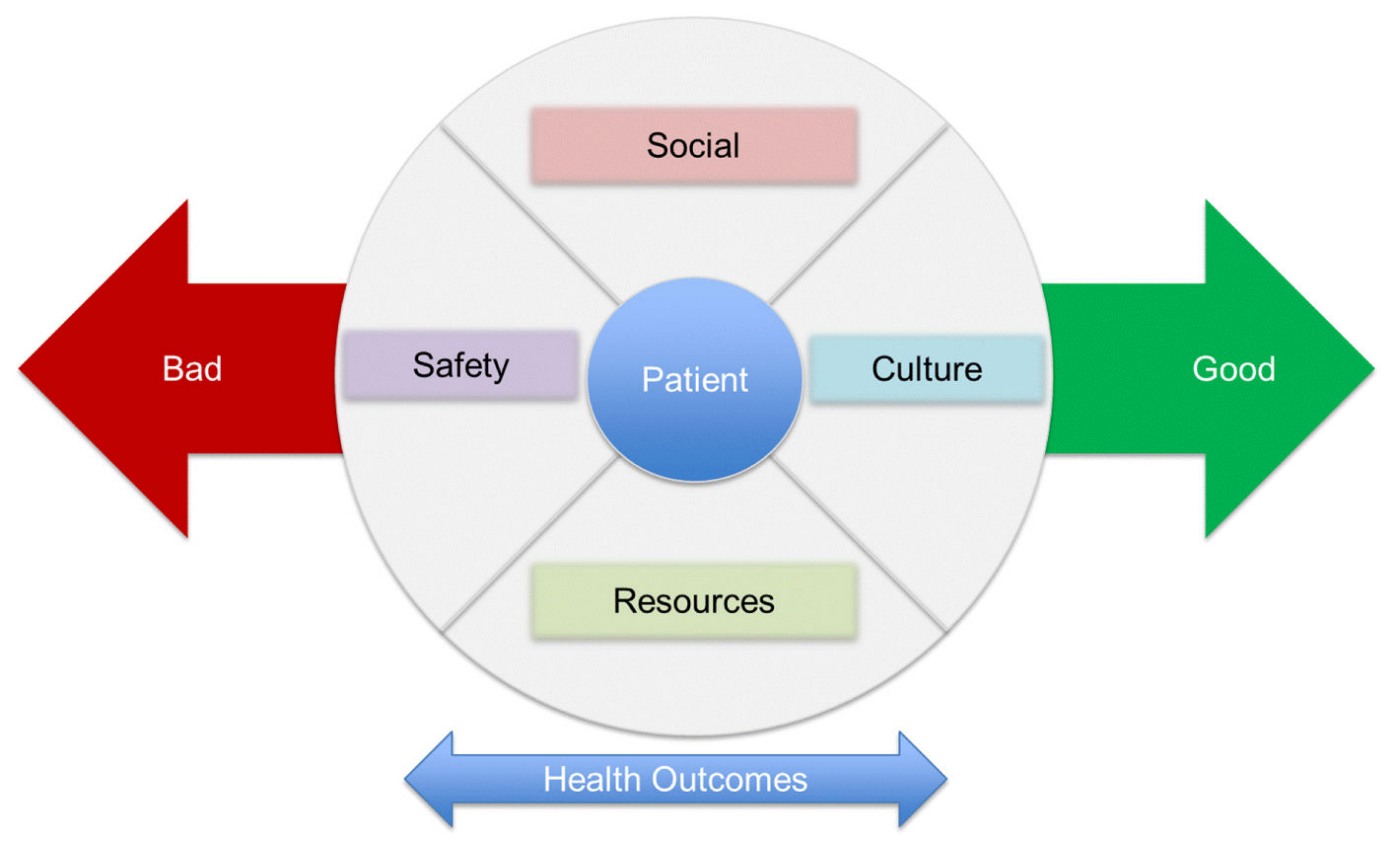
Circle of environmental influence on patient outcomes.

Patients from deprived communities have been found to have higher blood glucose levels and glycosylated hemoglobin (HbA_1c_) [11]. Patients are at the center of health interventions, but they reside in environments that impact their health outcomes; as such, our interventions to improve diabetic control must also take communities and neighborhoods into consideration. As demonstrated in Figure 1, patients may be moved along the continuum of health outcomes by individual systemic characteristics and by those found in their environments. This review describes 1) environmental risk factors related to diabetes and diabetes outcomes and 2) environmental risk factors for diabetic retinopathy.

## Contextual Framework to Assess Environmental Barriers

Social determinants of health are factors beyond intrinsic personal characteristics that influence the health of an individual [12]. Schulz and colleagues explored social determinants of racial disparities in diabetes risk in Detroit, a heavily racially segregated city with large areas of poverty [13]. Schulz identified four social determinants of health that mediated the relationship between race and the risk of diabetes: stressful life conditions, the built environment, the social environment, and educational opportunities. Stressful life conditions included measures of financial security and discrimination. The built environment described neighborhood safety, walkability, availability of fresh produce, and locations of fast-food restaurants. The social environment described social support for dietary practices and physical activity, workforce conditions, leisure time, and police capacity to maintain order. Educational opportunities included those that empowered community members to gain information about diabetes. By evaluating environmental and social risk factors for diabetic disease, Schulz and colleagues describe a sphere of influence beyond traditionally measured systemic risk factors. The East Side Village Health Worker Partnership created a community-level diabetes prevention program based on these findings. The identification of community-level modifiable risk factors enables change in health for an entire population.

Environmental factors act as barriers to diabetes prevention efforts and to diabetic care. Kieffer and colleagues designed community planning focus groups to discuss barriers to care with Black and Latino families in Detroit, Michigan [14]. Participants were aware of the morbidity and mortality associated with diabetes, including blindness and amputations, but they expressed concerns about barriers to optimal self-management including healthy eating and finding safe places to exercise within the environmental constraints of Detroit. Those that immigrated to Detroit reported both change in climate and lack of safety as barriers to healthy living. Barriers to healthy eating included limited financial resources, high cost of food, and family member preferences for different foods. These environmental barriers can negatively impact one’s ability to optimally manage his or her diabetes, especially in underserved populations. It is important to conduct environmental analyses to focus efforts to improve current environmental constraints, and ultimately, to improve outcomes for diabetic patients. If public health measures address some of these neighborhood-level barriers to diabetes management, then individuals become free to focus more on reducing their individual systemic risk.

## Analysis of the Environment

Studies that seek to demonstrate relationships between diabetes and the environment often utilize geographic information systems (GIS). Analytical programs create maps of environmental features with overlays of sociodemographic characteristics and available neighborhood resources at a level of granularity limited only by the data itself. Analyses determine how the distance between patients and necessary resources, such as screening locations, hospitals, outpatient facilities, pharmacies and grocery stores, impacts patients’ risk of developing diabetes. GIS analysis has been used to perform diabetic needs assessment and to focus on how to improve healthcare delivery [15–21]. It provides the geospatial context in which diabetic patients live, which is instructive in targeting treatment efforts.

Geraghty and colleagues evaluated primary care patients in California using GIS to assess whether or not the distance a patient travelled to their primary care doctor’s clinic was associated with diabetic control [22]. Sociodemographic characteristics were obtained from 2008 census tract level data on 7,288 patients and included median income, highest educational attainment, unemployment, and white or black race. While they did not find an association between the distances the subjects travelled to the clinics and diabetic control, their study did find a higher HbA_1c_ level in Black participants and in participants from lower income neighborhoods. While the difference in HbA_1c_ by race reflects known health disparities in diabetes, the effect of living in a low-income neighborhood effect was also significant. Though it is difficult to dissect the specific roles of each element of one’s environment, the results of this study underscore the need to do so.

Diez Roux and colleagues evaluated the association between insulin resistance and neighborhood characteristics [23]. Six components of the insulin resistance syndrome were evaluated in 5,115 young adults ages 18–30 in the ten-year Coronary Artery Risk Development in Young Adults (CARDIA) Study. The study included separate analyses of body mass index, fasting plasma high-density lipoprotein (HDL), triglycerides, insulin, and glucose levels as well as systolic blood pressure, which were combined into an insulin resistance syndrome score. The median household income, median home value, percentage of population earning interest income, percentage of population who completed high school, percentage of population who completed college, and the percentage of population in executive, managerial, or professional capacity were combined into a neighborhood socioeconomic score. The study found an association between the neighborhood score and insulin resistance syndrome, an important predictor of chronic disease. Addressing neighborhood-level barriers to care in young adults could lead to a healthier work-force with lower morbidity and mortality.

Auchincloss and colleagues evaluated insulin resistance on a neighborhood level in a non-diabetic population. Using person-level data from the Multi-ethnic Study of Atherosclerosis (MESA), the group evaluated insulin resistance defined as (fasting insulin (μU/mL) x fasting glucose (mmol/L))/22.5 in 2,026 participants [24]. The area level data was derived from the MESA Neighborhood Study, where participant neighborhood characteristics were taken from a community survey [25]. Insulin resistance was negatively correlated with residential environments that facilitated physical activity and where healthy foods were more widely available for purchase. The associations persisted after adjustment for age, sex, family history of diabetes, income, and education. After adjustment for race and ethnicity, the association with physical activity remained strong, but the associations with availability of healthy food were no longer significant. The authors suggested this may be related to the strong spatial patterning of type of food availability by race and ethnicity. This study demonstrates the impact of having an appropriate environment for physical activity and access to healthy food on precursor syndromes related to diabetes, which highlights the need for environmental interventions to decrease the number of people at risk for diabetes.

Other studies utilized GIS to evaluate screening and outreach programs. Kruger and colleagues evaluated survey data to facilitate the creation of a diabetes intervention in a high risk community in Genesee County, Michigan [19]. A point system was used to determine which individuals were at high risk for diabetes. Participants were surveyed about their diet, physical activity, health behaviors, overall health, and whether or not they had been screened for diabetes. Oversampling was conducted in zip codes defined as high risk areas based on previous assessment of health disparities in the region. Results demonstrated decreased report of screening rates in geospatially determined high risk areas, which allowed community organizations to focus efforts and resources on communities in need. It will be important to assess whether clinical outcomes, such as HbA_1c_ and complications related to diabetes, also vary by neighborhood and correspond to the same areas that report poor access to diabetes screening.

Curtis and colleagues used county-level diabetes and obesity data to map the prevalence of diabetes and diabetes-related resources [17]. Diabetes-related health information, population demographics, and diabetes resources and utilization were collected by county for the state of Michigan. County-level age adjusted obesity and diabetes data were derived from the Behavioral Risk Factor Surveillance System [26]. Diabetes resource availability and use were defined by the percentage of Medicare patients in the county that had HbA_1c_ tested within the study period, the number of endocrinologists per county, and available community resources. GIS was performed and counties with increased disease burden and lower resources were defined. The study was designed to detect an association between disease prevalence and resource availability; however, neither the GIS analysis nor the regression analysis found a significant association. While the study design was appropriate, a different set of variables may have demonstrated a different result. An analysis of primary care distribution may have been instructive, as many diabetic patients are not treated by specialists. In addition to the regression model, the authors mapped areas that had a high prevalence of disease, high percentage of minority population, and high poverty rate by county. An outcomes analysis overlying these descriptive analyses would be an important next step to guide diabetes screening program resource allocation.

Cravey and colleagues described a method by which to evaluate the acquisition of diabetic information in a rural community in North Carolina. They mapped socio-spatial knowledge networks of participant health beliefs about diabetes and physical places of knowledge acquisition [16]. These “social-spatial network nodes” were placed into categories based on the participant experience at a particular location, the availability of diabetes information, and how often participants visited a particular location. These data served as a basis for future focused community interventions. The next step would be to implement interventions at high volume locations to evaluate population-level outcomes in follow-up.

GIS facilitates a more targeted approach to managing diabetes on a population level, which can improve the cost-effectiveness of interventions by focusing on high-risk areas. Miranda and colleagues describe how data from Durham County was used to map 14,345 patients with ICD-9 codes for diabetes alongside HbA_1c_ levels, place of residence, billing, cost, environmental, demographic, community resources and birth and death records to create individualized and community-based intervention plans designed for a particular neighborhood context [27]. These analyses also allow for mapping the impact of interventions and providing quality assurance. Similarly, The Camden Coalition of Healthcare Providers built a database of patients that were seen in the emergency department over five years [15]. They determined that 20% of patients were responsible for 90% of the costs. A neighborhood-level analysis mapping claims data and neighborhood data with regards to diabetes was performed to define geographic areas of increased utilization and to provide a basis for the creation of patient-centered medical homes. A utilizer team was established to assist the most “expensive diabetic patients” and to design mobile outreach and new education platforms. These studies paired patient data and environmental data to create a change in care delivery in an attempt to increase the quality of care, while containing health care costs. Acknowledging the forces beyond each patient that impact his or her overall health is imperative in understanding how best to treat that patient. These studies are examples of integrated models which can decrease health care costs by focusing valuable resources on populations that need them the most.

## Environmental Factors in Diabetic Retinopathy

Diabetic retinopathy has not been evaluated nearly to the extent of systemic diabetes in a geospatial context. Weng and colleagues used GIS to evaluate morbidity and mortality in a diabetic population of 332 patients in London [28]. Social deprivation of each neighborhood was derived using the Jarman UPA score [29,30], which combines eight variables derived from census data including the percentage of elderly people living alone, percentage of one-parent families, percentage of children less than 5 years of age, percentage of social class V (unskilled workers), percentage of unemployed (as percentage of economically active population), percentage of overcrowded households, percentage of people changing household within the last year, and percentage of those born in the New Commonwealth or Pakistan. In this study, morbidity was evaluated clinically with neuropathy defined as absence of vibration perception, retinopathy defined as abnormalities on direct ophthalmoscopy, and proteinuria defined as a single positive urine protein. HbA_1c_, mortality, neuropathy, and proteinuria were all increased in socially deprived patients, while they did not find a statistically significant increase in the prevalence of retinopathy. In this study, a deprived environment was found to be associated with microvascular morbidity other than diabetic retinopathy; however, it is important to note that the measures for neuropathy and nephropathy were likely more sensitive than the limited view of the retina seen using direct ophthalmoscopy. In a study from Liverpool, Harding and colleagues found that direct ophthalmoscopy was only 65% sensitive for detecting sight-threatening diabetic eye disease, compared to 89% using fundus photography [31]. It is likely that the results for diabetic retinopathy were biased by the presence of false negatives. It would be important to assess the environmental impact on the prevalence of diabetic retinopathy using gold standard screening techniques such as Early Treatment Diabetic Retinopathy Study fundus photographs or a dilated fundus examination by an ophthalmologist.

Other studies have also evaluated environmental factors associated with diabetic retinopathy. Eachus and colleagues examined morbidity in a representative sample of 4,170 patients in the United Kingdom [32]. Questionnaires were co-signed by the primary care providers of the patients, completed by patients regarding morbidity related to overall health, and finally validated using medical records. The neighborhood impact was derived from postal codes and census data using the Townsend deprivation score [33], which incorporates percentage of unemployed, percentage that do not own a car, percentage that do not own a home, and household overcrowding. The study demonstrated an increased odds of diabetic eye disease (OR=3.21, 95% CI 1.84–5.59) among patients from the more deprived neighborhoods.

Scanlon and colleagues evaluated data from a mobile digital diabetic retinopathy screening program among over 10,000 patients in Gloucestershire in the United Kingdom. The sociodemographics of each patient were evaluated and their postal codes were used to determine the Indices of English Deprivation which includes income deprivation, employment deprivation, health deprivation and disability, education skills and training deprivation, barriers to housing and services, and crime and living environment deprivation [34]. Patients were divided into quintiles of deprivation. They showed an 11% decrease in screening uptake between quintiles (OR 0.89, 95% CI 1.08–1.15, p<0.001) and increased sight-threatening retinopathy in patients living in the most deprived areas (OR 0.95, 95% CI 0.90–0.99, p=0.02).

Leese and colleagues evaluated diabetic participant utilization of a well-established screening program in Scotland [35]. Census-based areas derived from postal codes and associated geographic data were used to define distance between participants and either an urban or rural screening location. Deprivation was defined using the Carstairs deprivation code: percentage of unemployed males over the age of 16, percentage of individuals in households with more than one person per room, percentage of households with no car, and the percentage of heads of households that were partially skilled or unskilled workers [36]. The analysis demonstrated how likely participants were to miss assigned screening. While the distance to the screening location was not a contributing factor, residing in a deprived area, young age, diabetic control, duration of diabetes, smoking status, higher blood pressure, and being invited to screen in a mobile van as opposed to a hospital all increased the likelihood that participants would miss screenings. Those that lived in the most deprived areas were 2.32 times (95% CI 1.92–2.81) more likely to miss screening, than those that lived in the least deprived areas. In addition, those who missed screenings were 3.13 times more likely to require subsequent laser (95% CI 1.58–6.18). These two screening studies demonstrated that patients in deprived areas were more likely to miss a screening appointment and when they presented, their disease was more advanced and they were more likely to need treatment. These studies on diabetic retinopathy and deprivation were done outside of the US. Diabetes is the leading cause of blindness in working age people in the US. It would be instructive to determine the impact of neighborhoods on outcomes in the US, where the disease burden is great and costly. The European studies described use deprivation scores to compare the socioeconomics of communities, but these studies lack the granularity of the built environment and miss some of the daily barriers that many patients face. Further research in the US should expand beyond deprivation scoring to include the broader environment.

## Conclusion

Risk factors for DR are complex, and initial work has demonstrated that the neighborhood environment in which patients live influences diabetes and microvascular complications from diabetes. Because of the high individual cost of blindness to patients and the high societal cost of diabetes-related eye care, it is imperative to design interventions that focus on controlling diabetes at both a community level and at an individual level. Patients are at the center of disease intervention, but we must not ignore the environments in which they live. As presented in Figure 1, outcomes are influenced by environment and may be pushed along the continuum by strategic cost-effective community-centered interventions. Medical interventions are patient centered, but patients are surrounded by other factors which may be influenced by available resources, safety, social situations, and/or cultural factors. Analysis of environmental factors provides a specific description of neighborhoods, which serves as a platform for both neighborhood-level interventions such as improving safety, walkability and access to healthy foods and individual-level interventions, such as increasing health system outreach and screening capacity for DR in higher risk neighborhoods. This type of targeted approach to improving diabetic control on a population level could lead to more effective deployment of health care resources and decreased morbidity for high-risk patients. Blindness is a significant morbidity for nearly 700,000 diabetic patients and the diabetic population continues to increase. It is paramount to involve environmental risk factor assessment in the creation of solutions to end blindness from diabetic eye disease.
